# Perspectives on Post–Stress Test Decision-Making and Preferred Outcomes Among Older Adults

**DOI:** 10.1001/jamanetworkopen.2025.29033

**Published:** 2025-08-26

**Authors:** Krishna K. Patel, Tansu Eris, Christina M. Pacheco, Kaesha Bowers, Parag Goyal, Fred Ko, Ksenia Gorbenko, Leslee J. Shaw, Laura P. Gelfman

**Affiliations:** 1 Department of Medicine, Icahn School of Medicine at Mount Sinai, New York, New York; 2 Department of Population Health Science and Policy, Icahn School of Medicine at Mount Sinai, New York, New York; 3 Department of Geriatrics and Palliative Medicine, Icahn School of Medicine at Mount Sinai, New York, New York; 4 Department of Family Medicine and Community Health, University of Kansas Medical Center, Kansas City; 5 Program for the Care and Study of the Aging Heart, Department of Medicine, Weill Cornell Medicine, New York, New York; 6 Geriatric Research, Education, and Clinical Center, James J. Peters VA Medical Center, Bronx, New York

## Abstract

**Importance:**

Among symptomatic older adults, noninvasive stress imaging is commonly used to assess for coronary artery disease. Comorbid geriatric conditions make an older adult population more vulnerable to complications of invasive testing and posttest treatment.

**Objective:**

To understand attitudes about post–stress imaging decision-making and preferred outcomes among older adults who had a stress test for suspected ischemic symptoms and their caregivers.

**Design, Setting, and Participants:**

In this qualitative study, focus groups were interviewed between July 2023 and March 2024. Focus groups included older adult patients (age ≥75 years) with recent cardiac stress imaging (within 12 months) at an urban academic hospital.

**Main Outcomes and Measures:**

The focus group facilitator guide addressed 3 domains: patient-physician communication during testing, patient preferences regarding decision-making and outcomes, and influence of geriatric impairments on decision-making. Thematic analysis used the constant comparative method to identify themes. Thematic saturation determined sample size.

**Results:**

A total of 8 focus groups were conducted with 29 participants (mean [SD] age, 79.0 [4.1] years; 17 [59%] women; 8 [28%] with ischemic stress test results). Regarding patient-physician communication, participants prioritized discussions of how age-related challenges might affect outcomes but felt that physicians rarely discussed these issues, provoking anxiety and confusion about test results. Participants desired more time, information, and support for informed decisions. Patients’ trust in their physicians influenced the role they wanted to play in medical decision-making. Most patients felt responsible for managing their health, and caregivers served as important health care advocates. Patients reported that multiple chronic conditions, changes in functional and mental health status, and a strong desire to maintain independence shaped health goals. Quality of life often outweighed longevity, although individual preferences varied.

**Conclusions and Relevance:**

In this qualitative study of older adults who recently underwent stress testing, participants described limited communication about age-related concerns, valued physician and caregiver input, and prioritized maintaining quality of life and independence. These findings suggest that integrating geriatric assessment and patient-defined goals into shared decision-making might help guide more patient-centered care in this population.

## Key Points

**Question:**

What are older adults’ perspectives regarding treatment decision-making and outcome preferences after cardiac stress imaging?

**Findings:**

In a qualitative study of 29 adults aged 75 years or older who recently underwent stress imaging, participants highlighted poor communication with physicians about age-related concerns, expressed a strong preference for quality of life over longevity, and trusted physician and caregiver input in decision-making.

**Meaning:**

These findings suggest that systematically integrating geriatric assessments and goal-setting conversations into shared decision-making after cardiac stress testing may improve care alignment and outcomes for older adults.

## Teaser

This qualitative study describes attitudes and preferences among older adults regarding decision-making and care outcomes after cardiac stress testing.

## Introduction

Approximately 1 in 4 adults aged 75 years or older who present with suspected ischemic symptoms have stable coronary artery disease (CAD).^1^ Cardiac stress imaging is frequently used in this population to guide diagnostic and therapeutic decisions.^2^ Following stress imaging, clinicians must often decide between intensifying medical therapy alone or pursuing further invasive evaluation, such as coronary angiography with potential revascularization. These posttest decisions can carry significant implications for older adults, who may experience higher procedural risks, longer recovery times, and uncertain benefits due to age-related physiological changes and coexisting geriatric conditions (eg, functional and cognitive impairments and multimorbidity).^3^

Coexisting geriatric syndromes can influence both the feasibility and outcomes of invasive and medical interventions. For example, frail or functionally impaired patients may be less able to tolerate invasive procedures or adhere to complex medication regimens. Moreover, the presence of geriatric conditions may shift the balance of risks and benefits, leading patients to prioritize other outcomes, such as symptom relief, preservation of independence, or quality of life, over longevity or anatomical completeness of revascularization. Despite the importance of these decisions, there is limited understanding of how older adults perceive the implications of stress test results and how they weigh their options in light of personal goals, health priorities, and age-related risks. Existing clinical frameworks often fail to account for the unique needs of this population, and treatment discussions after stress imaging may not adequately reflect what matters most to older patients.

To design more effective shared-decision-making strategies in this setting, it is important to first understand patient preferences, values, and perspectives after stress testing, when key CAD management decisions are being made. In this qualitative study, we aim to understand patients’ attitudes about decision-making after cardiac stress imaging, how age or geriatric conditions affect their posttest management decisions, and which outcomes they prioritize when considering their options for CAD management.

## Methods

This qualitative study was approved by the Mount Sinai institutional review board. All focus group participants provided informed consent prior to participation. This study is reported following the Consolidated Criteria for Reporting Qualitative Research (COREQ) reporting guideline.

We conducted focus groups with older adult patients (age ≥75 years) who underwent stress imaging (eg, stress echocardiography or stress nuclear myocardial perfusion imaging) to evaluate suspected ischemic symptoms at 1 academic community hospital in New York, New York, over a 12-month period. Inclusion criteria were age 75 years or older, underwent stress imaging (stress echocardiography, nuclear perfusion imaging or stress cardiac magnetic resonance imaging) for ischemic symptom evaluation within past 12 months, able to speak English, and able to consent for participation. We included patients referred for both pharmacologic and exercise stress imaging to capture a range of functional and symptom profiles. We used imaging databases and electronic health records to identify eligible patients. Patients were contacted by telephone and invited to participate in focus groups using purposeful sampling to ensure diverse representation across age, sex, race, education levels, and patient-reported stress test results (ie, nonischemic, ischemic, or unknown). Participants attended focus groups either using Zoom video conferencing software (Zoom Communications) or in person, based on preference.

Of 300 eligible patient participants, 29 patients agreed to participate. We conducted 8 patient focus groups. Prior to each focus group, participants completed a brief intake form that included self-reported demographic information, clinical history, and their understanding of their recent stress test result to help describe the characteristics of the focus group sample. Race and ethnicity were categorized as African American, Hispanic or Latino, or White. The focus groups were facilitated by trained moderators (T.E. or K.B.) and followed a structured moderator’s guide based on a phenomenological framework.^4^ The interview guide (eAppendix in Supplement 1) focused on 3 main themes, refined iteratively during the study: patient experiences discussing the need for stress tests, sharing results, and making subsequent treatment decisions with physicians; preferences for decision-making and desired health goals, including willingness to accept trade-offs in treatments; and the impact of age-related comorbidities and geriatric syndromes on treatment goals and decisions. All participants received $50 gift card for participation.

Focus group discussions were recorded and transcribed verbatim by a professional transcription service (Transcription Professionals). The study team (K.K.P., T.E., C.M.P., and K.G.) independently coded the transcripts and reviewed session notes concurrently with data collection using the constant comparative method.^5^ Transcripts were analyzed to identify specific patterns and themes, with formal coding applied to relevant statements in DeDoose software (SocioCultural Research Consultants). A taxonomy of themes was created to organize and refine the coding process. Regular virtual meetings were held to review individual coding, resolve discrepancies through consensus, and refine conclusions in the context of the complete dataset.

## Results

Patient characteristics are presented in Table 1. A total of 8 focus groups were conducted with 29 participants (mean [SD] age 79.0 [4.1] years; 17 [59%] women; 8 [28%] with ischemic stress test results). By race and ethnicity, there were 6 African American patients (21%), 2 Hispanic or Latino patients (7%), and 20 White patients (69%). All participants had at least 1 cardiovascular comorbidity, 20 participants (69%) had multiple (≥2) chronic conditions, and 10 participants (35%) had a history of CAD. By patient report, 18 patients (62%) had nonischemic stress test results, 8 patients (28%) had ischemic results, and 3 patients (10%) did not know. More than half of participants (16 participants [55%]) experienced no change in their management (ie, medication change and/or invasive referral) after the stress test , 9 participants (31%) reported medication adjustment after testing, 3 participants (10%) were referred for invasive angiography, and 4 patients (14%) were referred for another diagnostic test. The median (IQR) duration of the focus group sessions was 84 (70-91) minutes. Our analysis yielded 3 major themes: patient-physician communication around stress testing care; decision-making and roles of the patient, caregiver and physician; and preferred health care goals (Table 2).

**Table 1.** Characteristics of the Patient Focus Group Participants

Characteristic	Participants, No. (%) (N = 29)
Age, mean (SD), y	79.0 (4.1)
Gender	
Men	12 (41)
Women	17 (59)
Race and ethnicity	
African American	6 (21)
Hispanic/Latino	2 (7)
White	20 (69)
Education	
≤Eighth grade or less	1 (3)
Some HS or HS diploma	5 (17)
Vocational or Some college	5 (17)
≥4-y College degree	17 (59)
Living location	
Urban	27 (93)
Suburban	2 (7)
≥2 Chronic conditions )	20 (69)
Cardiovascular comorbidities	
Hypertension	19 (66)
Hyperlipidemia	15 (52)
	6 (21)
Chronic kidney disease	5 (17)
Prior myocardial infarction	5 (17)
Prior stent/bypass surgery	8 (28)
Prior stroke	2 (7)
Atrial fibrillation	3 (10)
Time since most recent stress test	
≤6 mo	16 (55)
>6 mo to 1 y	10 (35)
>1 y	3 (10)
Patient-reported stress test result	
Nonischemic	18 (62)
Ischemic	8 (28)
Do not know	3 (10)
Changes in patient management after stress imaging	
No change	16 (55)
Medication adjustment	9 (31)
Referral to angiography and/or stenting	3 (10)
Referral to another diagnostic testing	4 (14)

**Table 2.** Summary of Key Themes and Supporting Quotes

Theme and subtheme	Example quote
**Patient-physician communication in stress testing care**	
Lack of direct communication about age and its impact on treatment options	“As far as my cardiologist is concerned, I bring [my age] up more than he does because I want to get that out in the open.…”
	“No, age never entered [discussion], but I do know that age does affect a number of changes in your heart. I knew that already. I did not [talk about my age] with the doctor.”
	“Well, the age thing is always the silver cloud hanging over everything. But no one says anything unless I bring it up.”
Confusion about stress test results and treatment options and a desire for more support	“They didn’t give me any choices, they just said they wanted to do the test and install the pacemaker. They really didn’t tell me anything about any other options... Even now, this has been 8 months, either or 9 months later, I don’t really know—I know almost nothing about why this happened, and I really tried to find out.”
	“But they only have a limited amount of time to talk, so I always feel if I get into a real serious discussion, my particular situation and the particular options, and why this would be superior to that, I’m imposing on them an unreasonable burden.”
	“Tell me what that means. Does it mean I’m okay for the next 10 years, or do I need to do another stress test in 6 months?”
**Decision-making and roles of patient, caregiver, and physician**	
Trust in physicians influenced their preferred role in shared decision-making	“I didn’t have much input into the decision-making. I don’t think I would have been able to give a good opinion because I am not a medical person, and I don’t know whether or not it was indicated.”
	“I think I’m a very good patient, with great trust for the people who take care of me. I rely completely on them. I’m an easy person for that.”
Patient’s primary role in managing their health	“I’m really responsible for my overall health. I mean, the doctor is there to help me and to track test results and other performance, but I think it’s up to me to try to stay on track, and if the numbers, if something is off, then I have to try my best to get it back where it should be. I think that’s my responsibility. I mean, there are medications which I’m taking, but even with those, I still have to do certain things to keep the numbers where they should be.”
Important role of caregivers in health care decision-making	“But the thing is that I always have somebody with me so that I can… they can assess what’s being said as well as me, and this way we can talk back and forth.”
	“I would say my husband and my children, I have a son and a daughter, [and they] are very involved in tracking my health care, and my sister as well. They are all a bit on the worry side of things. and I’m less the worrier…. I would say they are involved and very helpful and add knowledge and care. I’m very lucky.”
**Preferred health care goals**	
Multiple chronic conditions, functional status, and mental health influence health priorities	“I can’t tell you how many people I’ve been referred to—urologist, endocrinologist, orthopedic, because of my broken bones and fractures, a pulmonary doctor because I’ve had pneumonia 3 times in my life, a brain doctor, that’s not the title, vein doctor, because I’ve surgery on both of my legs, and now everything, like I said, is falling apart, so it all is affected with the heart.”
	“I certainly don’t feel as though I had the stamina that I had previous to having this issue, say a year ago or so... I’m slower, but I’m walking.”
	“It changed a lot of my life. I’m used to having great stamina, a lot of energy, a fast pace when I’m working. I’m not the same anymore.”
	“Depression can make things feel worse, you know, like your pains and everything. Everything—your outlook…, I don’t want to rain on y’alls parade, but I don’t want to live longer. I’m a very defunct person; I’m very depressed a lot.”
Quality of life and maintaining independence vs longevity	“I don’t want anybody to have to take care of me, bathe me, or do any of those things. And I want to be able to do what I want to do when I can. Independence, I think that’s what it is.”
	“[I want] better quality of life versus living longer, because the more you live, the more things are going to pop up... What’s going to come is going to come anyway. So, I don’t know. I would think that to have better quality of life is more important.”
	“If I die tomorrow, I die healthy. To live without the quality of life, I personally don’t want that.”
Significant variability in individual health care goals	“My goals are to enjoy my life and keep on going on. Enjoy my life until God calls me home. I mean, enjoy my life as much as I could.”
	“My goals are, again, to stay healthy as long as I can. But by staying healthy, I’m talking about pain. Not just mobility, although that’s very important, but pain. And, like dementia, pain is something I am not interested in spending a long time, having a long-term relationship with.”
	“Increase mobility. I have a rollator. I live on the seventh floor of an elevator building…. My goal is first—I’m off of the rollator, although I haven’t been out in the street yet. I have to be careful. But I want to be stronger.”
	“I think my goals are to have a good quality of life and not so much focused on how long I live, but the quality of life for the time that I’m living.”
	“I’m looking forward to my stepson’s wedding in October, and I have family in California who I see whenever I can… I just want to keep doing that until I don’t do it anymore.”

### Patient-Physician Communication Around Stress Testing Care

#### Lack of Direct Communication About Age and Its Impact on Treatment Options

Multiple patients noted that their physicians did not routinely address how their age or age-related comorbidities might impact treatment decisions after stress testing. Several participants emphasized that they had to raise the topic themselves, indicating a perceived communication gap. One patient shared, “My doctors always said I seemed young for my age, but that doesn’t help me understand if I should be doing this test in the first place.” Another stated, “No, I haven’t had any discussions related to age. I don’t even know if they considered it.” These examples illustrate that age was either omitted from clinical conversations or only surfaced because patients initiated the topic. One patient shared that she brings up her age with her cardiologist so that it stays at the forefront of their decision-making, “I mean, if this is a procedure that’s not recommended for someone over a certain age..., I want to know about these things so that I can work around that.” This left some patients uncertain about whether their treatment paths were appropriately aligned with their age and overall health.

#### Confusion About Stress Test Results and Treatment Options, and a Desire for More Support

Many participants felt confused due to insufficient explanations about stress test results and available posttest treatment options. Some reported having adequate information, but others found themselves scouring the internet for supplemental information. A participant noted, “I don’t know if it was a result of the stress test; there always seemed to be a communication confusion between me, the doctor, and his team.” Another participant expressed, “I really wish for more guidance and more willingness to suggest this, the pros or the cons... Sometimes I find more information, if I may say, online... but I prefer to rely on the person who’s taking care of me.” Participants also expressed a need for more time and clearer communication with their doctors during appointments. One participant noted, “Everybody’s on a timer. You’ve got 15 minutes. How much can you do in 15 minutes with your doctor? You write everything down; you try to condense it because you know you’re on a short leash when you get there.”

### Decision-Making and the Roles of the Patient, Caregivers, and Physician

#### Trust in Physicians Influenced Their Preferred Role in Shared Decision-Making

Participants varied in how much autonomy they wanted, often linked to how much they trusted their physicians. A patient who trusted their physician remarked, “I kind of believe that ‘don’t tell me where to put a sofa and I won’t tell you how to run a blood test...’ I trust the pilot when he takes off in the plane that we’re going to get to where we’re supposed to go.” Another patient highlighted a challenge stating, “[Shared] decision-making is a little tricky as far as interacting with doctors. They tend to be a little aggressive about what they believe is the right procedure and recommendations.” Another patient shared, “I’m trying to take responsibility for making judgments about [my treatment]. I want to listen to my doctor; I’d rather have somebody else who knows everything says ‘this is what you should do,’ and I just trust them and go out and do it, and it works.”

#### The Patient’s Primary Role in Managing Their Health

Many patients saw themselves as responsible for their own health, including coordinating care and lifestyle changes. A participant emphasized: “Well, I’m primarily responsible for my own health... I just want to reiterate that I’m responsible overall. I do expect to have doctors who respond and help me.” Another participant echoed this challenge: “My role as a patient? I don’t know what to answer about that. Other than that, I’ve got to do everything on my own.” However, they often felt left to navigate health care decisions without sufficient guidance from clinicians. A patient-participant shared, “I agree, but I said that before: that I can’t get the information. The doctor has the information, and they’re not sharing it with me... I feel like I need to be in charge, but I feel that it’s all I can do to find out the information.”

#### Important Role of Caregivers in Health Care Decision-Making

Participants mentioned the importance of having a caregiver present during medical appointments to assist them with comprehending medical information. Patients viewed their family members, friends, and caregivers as advocates who ensure that their needs and preferences are communicated to health care practitioners. One participant shared, “I always take someone with me to my appointments… because sometimes I do get a little forgetful, you know, with a lot of information. But my daughter is there as a backup, and she gathers all the information as well as I do.”

### Preferred Goals of Care

#### Multiple Chronic Conditions, Functional Status, and Mental Health Influence Health Priorities

For many participants, multiple comorbidities created a complex health profile requiring careful management and frequent monitoring. These comorbidities exacerbated physical symptoms and contributed to a noticeable decline in stamina and mobility. As one participant shared, “I have diastolic dysfunction, and I’m short of breath a lot. Part of it is my weight. Part of it is the asthma. But I also have diastolic dysfunction. So I’ve had several stress tests.” Others described frustration and sadness over losing prior levels of function: “I continue to get short of breath walking half a block, and I’m really disappointed about that because, like I said, I used to walk everywhere. That was my 1 saving grace that I believe helped me for years. Now in the last 10 years I’m not able to do that, and that does make me sad.” In addition to physical limitations, participants reported significant emotional challenges, including depression, anxiety, and social isolation, that impacted their overall well-being. Mental health emerged as a core component of how they defined quality of life. One participant recognized, “I’m an over-worrier, too... I’m addicted to worrying. I’m always asking questions. That can be detrimental.” Another reflected on the toll of loneliness and the importance of staying engaged: “Especially at our age, when we don’t have kids, our kids don’t have time for us... I get very, very depressed... I’ve found that if I just put my mind on something else, like I said, to volunteer or find something to do, because we get depressed, we’re alone.” For some, this led to a sense of hopelessness and diminished interest in prolonging life. These insights underscore that both emotional and functional well-being significantly influenced participants’ goals and preferences regarding further testing and treatment.

#### Prioritizing Independence and Quality of Life Over Longevity

Participants desired to maintain independence, engage in meaningful activities, and avoid prolonged suffering, even if it might mean a shorter lifespan. Many patients expressed a clear preference for quality of life, characterized by independence, the ability to engage in activities they enjoy, and the avoidance of pain and suffering, over living longer. A participant noted, “Quality of life, I think that means mostly to be independent as much as possible and not having to depend on others to live my life. And be able to do things that I enjoy doing, as much as possible, to not be prevented from doing things that I enjoy.” There was a clear aversion to interventions that could prolong life but diminish its quality, such as extensive medication regimens or invasive treatments. One patient shared, “I was offered a choice between medication and lifestyle, and I chose lifestyle because I am not going to be around, and I didn’t want any medication that was going to have side effects.” Another patient commented on their approach to health care, “I’ve made health decisions totally relevant to my quality of life, even if it shortens my life at some point. I’m very grateful that I’m healthy, so far.”

#### Significant Variability in Individual Health Care Goals

Participants in the study expressed a wide range of health care goals, reflecting their personal priorities, health conditions, and life circumstances. These goals varied from maintaining or improving physical and mental health to managing chronic conditions and reducing reliance on medications. One participant emphasized the importance of staying active: “The goal is to try and get back as much athletic ability as I had... My goal is to see how good, how athletic I can be with this situation.” Another participant highlighted cognitive health: “My goals would be to keep a quality of life, which I consider to be mentally and cognitively sharp enough... I want to avoid a stroke, another heart attack, anything that would diminish my mental abilities.” Maintaining a high quality of life and independence was a primary concern for many participants, as described at length above in regards to prioritizing those over longevity. Several participants discussed their goals related to managing chronic conditions and reducing their dependence on medications. One participant was clear about this goal, “…how can I get off this medication? I don’t want to take all these pills; I want to get rid of stuff.” These differences highlight the importance of individualized care plans aligning with each patient’s unique context and wishes.

## Discussion

In this qualitative study of adults aged 75 years and older who underwent cardiac stress imaging, we identified several insights into their posttest decision-making processes and the outcomes they most valued. Overall, participants wanted more discussions about how their age, multimorbidity, and geriatric syndromes might influence risks and benefits of further posttest interventions. However, few participants recalled having such conversations with their cardiologists or primary care physicians. Participants emphasized the responsibility for managing their own health but noted insufficient guidance in making decisions. These findings highlight the persistent gap between shared decision-making best-practices and the actual clinical experience of many older patients.

The diversity in health goals we identified reinforces that clinicians should not assume all older adults share uniform goals but rather must engage in in-depth, patient-tailored conversations to identify and align decision-making to patient priorities, such as those proposed in the Patient Priorities Care Framework.^6^ Our data align with prior work showing that older adults often prioritize quality of life, including daily function and independence, over quantity of life or longevity.^7,8^ However, our study showed that these outcomes are rarely discussed in post–stress test decision-making, highlighting the need for more individualized goal-based communication..

The lack of discussion of age-related concerns or health priorities by clinicians may be due to lack of time, limited training in geriatrics, or uncertainty regarding who is responsible for initiating these conversations, particularly in the subspecialty settings. To improve care alignment, we recommend incorporating brief targeted geriatric screening tools rather than comprehensive geriatric assessments in specialty care clinical pathways. Simple screens for frailty, cognitive impairment, or functional limitations during follow-up visits could serve as a practical starting point for identifying vulnerabilities and guide risk-benefit trade-off discussions. For patients with positive screening results, referral to geriatrics for a more comprehensive evaluation may support goal-concordant care. We also recognize that cardiologists may defer certain decisions to primary care clinicians or geriatricians, making interdisciplinary coordination essential. Development and use of decision aids specifically made for older adults for post–stress imaging decision-making and inviting caregivers to key appointments may further strengthen shared decision-making. The Box outlines practical, patient-informed strategies to support communication and decision-making for older adults in this setting.

Box. **Recommendations on Improved Communication Strategies After Stress Testing in Older Adults**

Discuss impact of age and geriatric syndromes as part of the decision-making discussion
Use plain language to explain test results and management options
Allocate dedicated time for shared decision-making discussions, where feasible
Identify responsible care team member for follow-up discussions to decrease confusion
Incorporate geriatric screening (eg, frailty, cognition, function) into posttest evaluation
Invite caregivers or trusted family members to participate in key discussions
Elicit patient health goals early in discussion (eg, symptom relief vs longevity)
Use decision aids tailored to older adults’ values (eg, quality of life, independence)

### Limitations

This study has some limitations. While our purposeful sampling and qualitative approach added depth, our modest 10% response rate, single-center nature, and limited sociodemographic diversity may affect generalizability. We were unable to collect detailed information on nonparticipants, which may limit our ability to fully assess the representativeness of our findings. We did not include physicians, whose perspectives might have added valuable context to these findings. Future work should explore how cardiologists, primary care physicians, and geriatricians perceive their roles in addressing age-related vulnerabilities and patient priorities, particularly within time-limited visits and evolving reimbursement models. Understanding clinician perspectives will be critical to designing feasible, scalable strategies that support individualized care for older adults.

## Conclusions

In this qualitative study of older adults who recently underwent stress testing, participants reported limited discussion of age-related factors, valued communication and caregiver input, and often prioritized quality of life and independence over longevity. Integrating brief geriatric screening and patient-defined goals into post–stress test discussions may help promote more individualized, patient-centered care in this vulnerable population.

## Notes

Supplement 1. **eAppendix.** Focus group interview guide

Supplement 2.
Data Sharing Statement
