# Supplementary material for: Perspectives on Post–Stress Test Decision-Making and Preferred Outcomes Among Older Adults
Source: JAMA Netw Open. 2025 Aug 26;8(8):e2529033. doi: 10.1001/jamanetworkopen.2025.29033 (PMC12381669; doi:10.1001/jamanetworkopen.2025.29033)
Supplement: Supplement 1. — eAppendix. Focus group interview guide [file jamanetwopen-e2529033-s001.pdf]

Supplemental Online Content

Patel KK, Eris T, Pacheco CM, et al. Perspectives on post–stress test decision-making and preferred outcomes among older adults. *JAMA Netw Open*. 2025;8(8):e2529033. doi:10.1001/jamanetworkopen.2025.29033

eAppendix. Focus group interview guide

This supplemental material has been provided by the authors to give readers additional information about their work.

© 2025 Patel KK et al. *JAMA Network Open*.

Supplement 1: Focus group interview guide

1. **To get started, please introduce yourself with your first name and share why did you get a stress test and what did it show?**

Probe (optional): Did your doctor discuss any other options to the stress test with you when they ordered it?

Probe (optional): Was an option of starting or changing medications before a stress test discussed with you?

Probe: How involved were you in the decision making to get a stress test?

1. **Tell us how your doctor discussed the results of the stress test with you?**

(use explanation only if needed: did they explain what options for different treatments you have such as medicines, angiogram/cath, stent, both, etc.)

2. **Did your doctor ever discuss how your age and age-related problems such as physical weakness, memory or balance issues would affect how you would respond to stress test or treatment after stress test such as medicines or angiogram etc.?**

(Probe: if yes, can you please describe what this conversation was like?)

Next 2 questions: use if pts test was abnormal and they had some treatment offered to them such as medicines or angiogram etc.

a) **What were the reasons you had for agreeing to choose your treatment option?**

   (use next only if they need to understand Q better: *Such as one having less side effects, one not impacting the quality and were they the final decision or were other treatments eventually chose?)*

b) **Were side effects and the possibility of other problems occurring for different txs discussed?**

   (only if needed: *Such as a medication versus a stent increasing weakness, frailty, memory issues, balance problems?)*

   Probes: Who chose your treatment? Did you agree with this? Was the treatment decision final?

c) Is there any other information you wanted that you wish was discussed with you? *(only if needed: such as receiving more information on the meaning of your results or the pros and cons of each tx option)*

7. If you have been diagnosed with coronary artery disease (blockages in the blood vessels of your heart), how has it affected your life?

© 2025 Patel KK et al. *JAMA Network Open*.

8. Have you ever had a conversation about your health care goals with your doctor?

If yes, can you please describe it?

Probe: What would be the best scenario for such conversations? Such as before or after a stress test, or some other scenario?

Probe: Who initiated the conversation?

9. (If not discussed above) What are your healthcare goals? (such as living longer, living w/o pain, *Preventing complications***)**

Probe: Alternative question/probe (if initial Q is unclear): **When making decisions about managing your heart disease, what outcomes or results are most important for you?** (such as *Removing blockages,* decreasing depression, *Reducing care giver burden to family, better quality of life, others like this*) *Continuing your life like you have it now, avoiding stress, depression, anxiety or Improving symptoms of chest pain and shortness of breath?*

10. **How do you view your role as a patient in your overall health and as a patient with coronary heart disease. More specifically, how you see your role when it comes to decision making and getting information about your disease from your doctor.** *(ok to skip if time constraint).*

    Optional (skip if less time): **When it comes to obtaining information, do you see yourself as your primary advocate or do you see your doctor as support and provide you with a sufficient amount of information, such as with dx causes, test results, and tx options?**

    Optional (skip if less time): **Do you think your dr should provide you with more information? If so, what kind and how much?**

11. **Is there anything else I should ask so I can better understand how you deal with your coronary artery disease?**

© 2025 Patel KK et al. *JAMA Network Open*.
