# Supplementary material for: Perspectives on Post–Stress Test Decision-Making and Preferred Outcomes Among Older Adults
Source: JAMA Netw Open. 2025 Aug 26;8(8):e2529033. doi: 10.1001/jamanetworkopen.2025.29033 (PMC12381669; doi:10.1001/jamanetworkopen.2025.29033)
Supplement: Supplement 2. — Data Sharing Statement [file jamanetwopen-e2529033-s002.pdf]

Data Sharing Statement

Patel. Perspectives on Post–Stress Test Decision-Making and Preferred Outcomes Among Older Adults. *JAMA Netw Open*. Published August 26, 2025.
doi:10.1001/jamanetworkopen.2025.29033

Data
Data available: Yes
Data types: Deidentified participant data
How to access data: On request to corresponding author at Krishna.Patel5@mountsinai.org, under a data use agreement between institution.
When available: With publication

Supporting Documents
Document types: None

Additional Information
Who can access the data: Researchers whose proposed use of the data has been approved by IRB; and under a data use agreement
Types of analyses: qualitative analysis
Mechanisms of data availability: signed data access agreement
Any additional restrictions: no restrictions
